# Retromandibular Anteroparotid Versus Transparotid Approach for Subcondylar Mandibular Fractures: A Retrospective Comparative Study of 80 Cases

**DOI:** 10.3390/jcm15020887

**Published:** 2026-01-21

**Authors:** Andrea Battisti, Danilo Di Giorgio, Federica Orsina Ferri, Marco Della Monaca, Benedetta Capasso, Paolo Priore, Valentina Terenzi, Valentino Valentini

**Affiliations:** 1 Department of Oral and Maxillofacial Sciences, Sapienza University of Rome, Via Caserta 6, 00161 Rome, Italy; 2 Unit of Maxillofacial Surgery, Azienda Ospedaliero-Universitaria Policlinico Umberto I, Viale del Policlinico 155, 00161 Rome, Italy; 3 Department of Surgical Sciences, Tor Vergata University, 00133 Rome, Italy

**Keywords:** subcondylar fractures, retromandibular approach, anteroparotid access, open reduction and internal fixation (ORIF), facial nerve preservation, maxillofacial trauma

## Abstract

**Background/Objectives**: Subcondylar mandibular fractures represent a challenging subset of maxillofacial trauma due to their proximity to the temporomandibular joint and the facial nerve. The retromandibular approach can be performed through either an anteroparotid or a transparotid route, but comparative clinical data remain limited. This study aimed to evaluate clinical outcomes, complication profiles, and operative parameters associated with the retromandibular anteroparotid versus transparotid approach for open reduction and internal fixation (ORIF) of subcondylar fractures. **Methods**: A retrospective analysis was conducted on 80 consecutive patients treated for subcondylar mandibular fractures at the Department of Maxillofacial Surgery, Umberto I General Hospital, Sapienza University of Rome, between 2018 and 2025. All patients underwent ORIF via a retromandibular approach (anteroparotid or transparotid) with a minimum follow-up of 6 months. Demographic data, trauma etiology, fracture morphology (classified as simple or complex), associated fractures, surgical approach, fixation details, operative time, hospital stay, and postoperative complications were collected. Facial nerve function was clinically assessed and graded using the House–Brackmann scale. Associations between fracture type, surgical approach, number of plates, and complications were evaluated using Chi-square or Fisher’s exact tests, while operative time was compared using one-way ANOVA and Kruskal–Wallis tests (*p* < 0.05). **Results**: The cohort had a mean age of 41.9 years and was predominantly male (67.5%). The anteroparotid route was used in 54 patients (67.5%) and the transparotid route in 26 (32.5%). Overall, 10 patients (12.5%) developed postoperative complications, including transient facial nerve weakness, malocclusion, visible scarring, and sialocele. All cases of sialocele occurred in the transparotid subgroup, whereas no salivary complications were observed after the anteroparotid approach. No permanent facial nerve deficits, temporomandibular joint ankylosis, or long-term facial asymmetry were recorded at 6 months. No significant association was found between surgical approach and overall complication rate, but complex fracture patterns were significantly associated with increased operative time. **Conclusions**: The retromandibular approach is a safe and effective option for ORIF of subcondylar mandibular fractures. Both anteroparotid and transparotid routes provided reliable exposure and stable fixation with low complication rates. The anteroparotid route appears to minimize parotid-related complications, such as sialocele, while maintaining comparable functional outcomes. These findings support the retromandibular anteroparotid approach as a valuable alternative in the surgical management of subcondylar fractures.

## 1. Introduction

Condyle fractures represent a significant percentage of mandibular fractures, occurring in 20–52% of cases [1,2]. Given the high frequency of these fractures, defining common, evidence-based therapeutic strategies is crucial. This is particularly important as undiagnosed or incorrectly treated condylar fractures can lead to severe functional impairments, including poor occlusion, restricted mouth opening with associated deviation, and limited lateral mandibular movement. Additionally, fractures with major displacement may result in shortening of the posterior facial height, leading to significant asymmetry [3,4]. Although numerous classifications exist, a simpler framework differentiates condylar fractures into intracapsular and extracapsular types. Considering the extracapsular types, the treatment of subcondylar fractures remains a subject of ongoing debate among maxillofacial surgeons [5], as outcomes following treatment with various techniques are often not consistently interpretable. Currently, therapeutic decisions continue to evolve, with the literature progressively emphasizing the superiority of surgical treatment over conservative approaches [3] even though the choice of surgical method is still often influenced by the surgeon’s technical expertise. The main therapeutic options currently available include open reduction with internal fixation (ORIF), which can be performed through submandibular or retro-mandibular approaches, either anteroparotid, transparotid and retroparotid or intraoral access with endoscopic assistance and internal fixation [6]. Conservative management with mandibulo-maxillary fixation (MMF) may also be employed, often in conjunction with other approaches, to ensure a safer healing process by maintaining the patient in the correct occlusal position [7]. However, all surgical options come with distinct advantages and drawbacks. Extraoral approaches carry the risk of permanent or temporary facial nerve injury and may result in visible scarring. Transoral access avoids these complications but presents greater technical challenges, as it does not provide a wide view of the surgical site. Furthermore, MMF may facilitate easier stabilization of the fracture, but it is associated with significant patient discomfort and, in some cases, temporomandibular joint (TMJ) ankylosis [8]. Despite the widespread use of the retromandibular approach for the surgical management of subcondylar mandibular fractures, direct comparative evidence between its anteroparotid and transparotid variants remains limited. Most available studies report small, heterogeneous case series or focus on a single surgical corridor, making it difficult to draw robust conclusions regarding differences in clinical outcomes and complication profiles. In particular, data directly comparing parotid-related morbidity, facial nerve outcomes, and operative parameters between the two approaches within a homogeneous surgical setting are scarce. The present study addresses this gap by retrospectively analyzing a consecutive cohort of 80 patients treated at a single tertiary referral center, all managed with open reduction and internal fixation via a retromandibular approach. By directly comparing the anteroparotid and transparotid routes within the same institutional protocol, this study aims to provide a more reliable assessment of their relative safety, reproducibility, and clinical performance.

## 2. Materials and Methods

This retrospective cohort study included all consecutive patients treated for subcondylar mandibular fractures at the Department of Maxillofacial Surgery, Umberto I General Hospital, Sapienza University of Rome, between January 2018 and December 2025. The study was conducted in accordance with the Declaration of Helsinki and was approved by the Institutional Review Board of Sapienza University of Rome (Prot. n. 0001107N.6/2024).

Inclusion criteria were: (1) radiologically confirmed subcondylar mandibular fracture; (2) primary treatment with open reduction and internal fixation via a retromandibular approach (anteroparotid or transparotid); (3) availability of complete clinical and radiological documentation; and (4) a minimum follow-up of 6 months. Exclusion criteria included fractures located outside the subcondylar region, patients managed with closed treatment only, and patients lost to follow-up before 6 months. Associated mandibular or facial fractures (e.g., symphyseal, parasymphyseal, or contralateral condylar fractures) were not considered exclusion criteria and were recorded for subgroup analysis.

All patients underwent preoperative evaluation consisting of clinical examination and imaging with either computed tomography (CT) or panoramic radiography to assess fracture pattern and extent. The following clinical and surgical data were collected: age, sex, etiology of trauma, type of fracture, surgical approach, operative time (minutes), length of hospital stay (days), type of fixation (internal or external), number of fixation plates, and postoperative complications.

Subcondylar fractures were first classified morphologically as monofocal, bifocal, trifocal, or comminuted based on preoperative CT or panoramic radiography. For comparative purposes, fractures were further grouped into simple (single fracture line) and complex (≥2 fracture lines, including bifocal, trifocal, and comminuted patterns).

All procedures were performed under general anesthesia using a retromandibular approach. A vertical skin incision of approximately 2–3 cm was placed 0.5–1 cm posterior to the posterior border of the mandibular ramus, below the earlobe. Two variants of the retromandibular approach were employed: (I) Transparotid route: after incision of the subcutaneous tissue and superficial musculoaponeurotic system, the parotid capsule was incised and blunt dissection was carried out through the parotid parenchyma, carefully identifying and preserving branches of the facial nerve. The masseter muscle was then split to expose the subcondylar region. (II) Anteroparotid route: the dissection plane was developed anterior to the parotid gland. After elevating the skin and subcutaneous tissue, the parotid capsule was retracted posteriorly and the facial nerve branches were identified or protected using blunt dissection. The surgical corridor was created through the masseter muscle without transgressing the parotid parenchyma, thereby minimizing the risk of salivary complications, such as sialocele.

In both approaches, the fracture site was exposed under direct vision, reduced, and fixated with titanium miniplates and monocortical screws following principles of rigid internal fixation. The use of one, two, or three plates was decided intraoperatively according to fracture stability and comminution. Maxillomandibular fixation was used selectively as an adjunct to ensure occlusal stability during fixation. The choice between anteroparotid and transparotid routes was based on fracture morphology, displacement pattern, soft tissue condition, and surgeon preference. In general, the transparotid route was favored in cases with marked medial displacement or when a more direct, short corridor to the fracture was required, whereas the anteroparotid route was preferred to avoid transgression of the parotid parenchyma in patients at higher risk for salivary complications (e.g., obese patients, patients with previous parotid surgery or ductal abnormalities).

Postoperative complications were systematically recorded and classified as: transient or permanent facial nerve dysfunction, malocclusion, temporomandibular joint (TMJ) ankylosis, facial asymmetry, and visible scarring. Postoperative facial nerve function was assessed clinically at each follow-up visit. Branch-specific motor function (frontal, orbital, zygomatic, buccal, marginal mandibular, and cervical) was evaluated and graded according to the House–Brackmann facial nerve grading system (grades I–VI). Facial nerve palsy was classified as transient if full recovery (House–Brackmann grade I) occurred within 6 months, and permanent if any residual deficit persisted beyond 6 months. The primary outcome measures were postoperative complications, including transient or permanent facial nerve dysfunction, malocclusion, sialocele, visible scarring, temporomandibular joint ankylosis, and facial asymmetry. Secondary outcomes included operative time and length of hospital stay. Outcomes were analyzed according to fracture complexity (simple vs. complex) and surgical route (anteroparotid vs. transparotid).

Descriptive statistics were used to summarize demographic and clinical variables. Categorical data were compared using Chi-square or Fisher’s exact test, as appropriate. Continuous variables were compared using one-way ANOVA and the Kruskal–Wallis test when non-normality was suspected. A *p*-value < 0.05 was considered statistically significant. Statistical analyses were performed using IBM SPSS Statistics, version 28.0.1.1 (IBM Corp., Armonk, NY, USA).

## 3. Results

**Figure 1:**
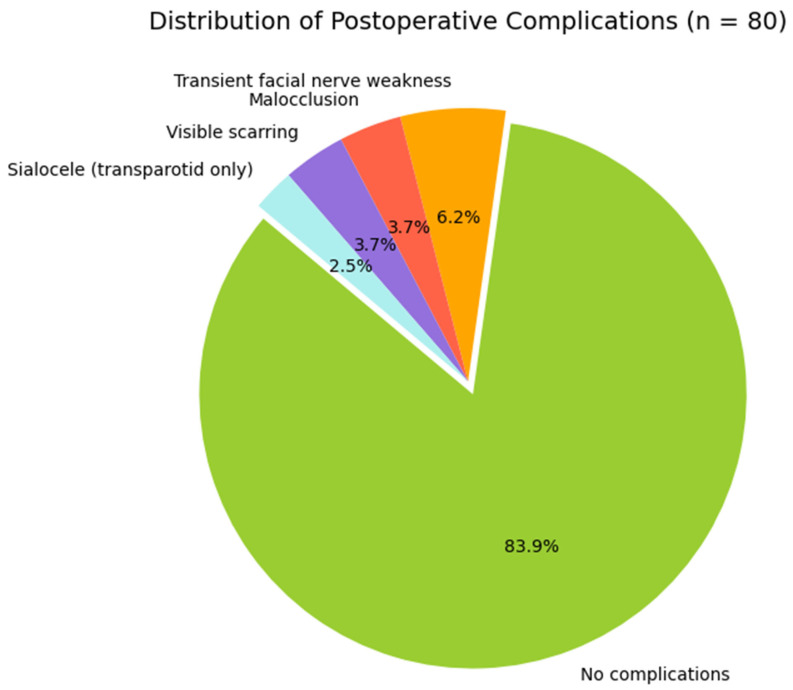
Distribution of postoperative complications among 80 patients treated for subcondylar mandibular fractures. The chart highlights the predominance of uneventful outcomes (83.9%), with transient facial nerve weakness (6.2%), malocclusion (3.7%), visible scarring (3.7%), and sialocele (2.5%) representing minor complications. Notably, all sialoceles occurred in patients treated via the transparotid route. The ‘No complications’ segment is exploded to emphasize the overall safety of the procedure.

**Figure 2:**
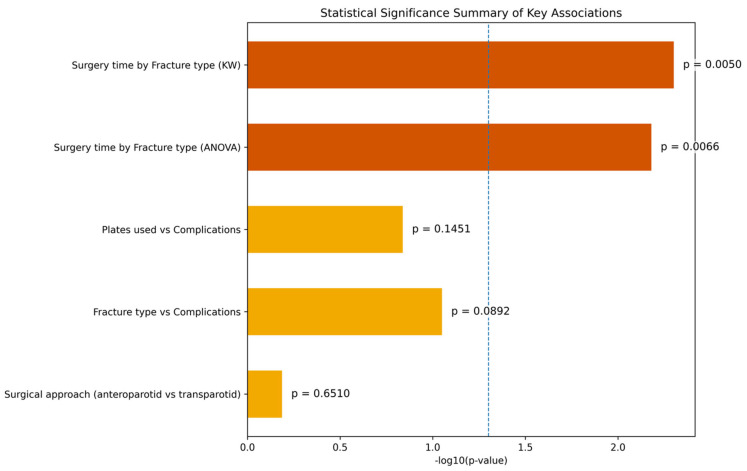
Summary of statistical significance for key variable associations. Bar height represents the negative logarithm of the *p*-value (−log_10_[*p*-value]) for each statistical test. The blue dashed line marks the conventional threshold of statistical significance (*p* = 0.05). Chi-square tests were used to evaluate associations between fracture type and postoperative complications, and between the number of fixation plates and complications. A one-way ANOVA and a non-parametric Kruskal–Wallis test both revealed a statistically significant difference in mean surgical time across fracture types (*p* = 0.0066 and *p* = 0.0050, respectively). No statistically significant difference was found between anteroparotid and transparotid approaches in terms of complication rate (*p* = 0.6510). Bars are colored according to significance: dark orange for statistically significant tests (*p* < 0.05), light orange for non-significant.

**Figure 3:**
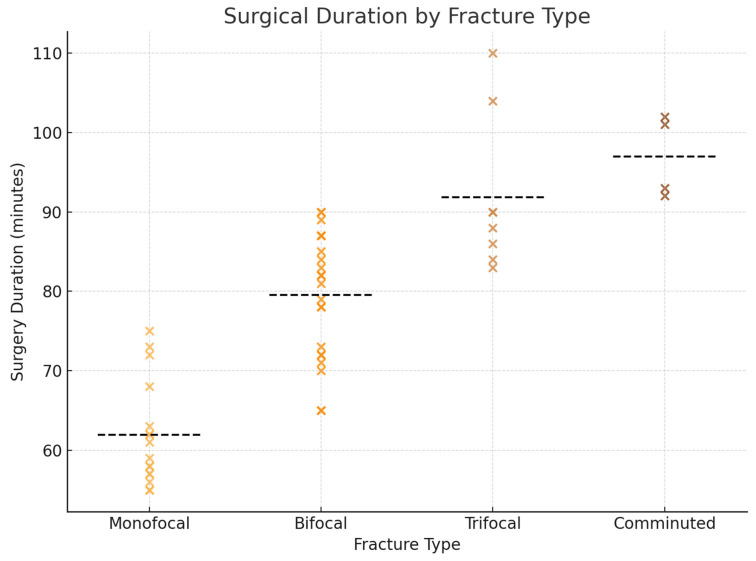
Scatter plot illustrating the distribution of surgical durations (in minutes) according to fracture type in patients undergoing open reduction and internal fixation of subcondylar mandibular fractures. Each cross represents an individual case. Horizontal dashed black lines indicate the mean operative time within each fracture category. A clear trend toward increased surgical time is observed in more complex fracture types, with monofocal fractures averaging the shortest durations and comminuted fractures the longest. This association was statistically significant (one-way ANOVA: *p* = 0.0066; Kruskal–Wallis test: *p* = 0.0050).

A total of 80 patients were included in this study. The mean age of the cohort was 41.9 years, and most patients were male (67.5%). The main etiologies of trauma were road traffic accidents and interpersonal violence, each accounting for 38% of cases, followed by accidental falls (24%). Regarding fracture morphology, bifocal fractures were the most common pattern (44%), followed by monofocal (32%), trifocal (16%), and comminuted fractures (8%). With respect to anatomical distribution, the combination of a subcondylar fracture with a symphyseal fracture represented the most frequent configuration (34%), while isolated subcondylar fractures were observed in 28% of patients. Less common patterns included subcondylar fractures associated with parasymphyseal involvement or with symphyseal and contralateral condylar fractures (both 6%), as well as subcondylar fractures combined with a contralateral condylar fracture alone (8%) (Table 1). All patients underwent open reduction and internal fixation via a retromandibular approach. In most cases (67.5%) an anteroparotid route was used, whereas the transparotid route was employed in the remaining 32.5%. Fixation was performed with titanium miniplates and monocortical screws, usually with two plates, although the number varied from one to three depending on fracture complexity. The mean operative time was 84.4 min (range: 55–120), and the average postoperative hospital stay was 5.3 days (range: 2–9). Although age may influence fracture patterns and healing, the sample size did not allow for reliable age-stratified subgroup analyses. Therefore, age was considered as a covariate rather than a grouping variable. Postoperative complications occurred in 10 patients. Transient facial nerve weakness was the most common complication (6.2%), followed by malocclusion (3.7%) and visible scarring (3.7%). Two cases of sialocele (2.6%) were recorded, both of which occurred in patients treated through a transparotid approach. No cases of permanent facial nerve injury, temporomandibular joint ankylosis, or long-term facial asymmetry were observed at the six-month follow-up (Figure 1). The comparison between the anteroparotid and transparotid routes did not reveal statistically significant differences in the overall complication rate or in the incidence of specific complications, including facial nerve weakness, malocclusion, scarring, or sialocele formation, even though the latter was observed exclusively in the transparotid group (Figure 2). Analysis of operative time showed a significant association with fracture complexity, with trifocal and comminuted fractures requiring longer surgical times. This was confirmed by both one-way ANOVA (F = 5.04, *p* = 0.0066) and Kruskal–Wallis testing (H = 10.39, *p* = 0.0050) (Figure 3). In contrast, no significant relationship was identified between fracture type and postoperative complications (χ^2^ = 16.36, *p* = 0.0892), nor between the number of plates used for fixation and the occurrence of complications (χ^2^ = 15.35, *p* = 0.1451).

## 4. Discussion

**Figure 4:**
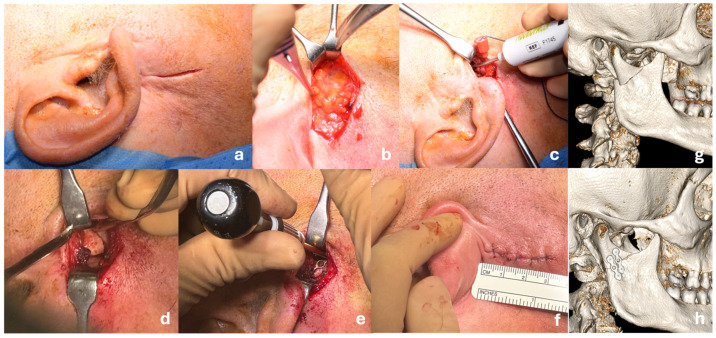
Intraoperative sequence and 3D imaging illustrating the retromandibular approach for open reduction and internal fixation of a subcondylar mandibular fracture. (**a**) Preauricular preparation and skin incision with identification of the incision site behind the mandibular border; (**b**) Blunt dissection through the subcutaneous and parotid layers, creating the transparotid or anteroparotid surgical corridor; (**c**) The dissection proceeds through the fibers of the masseter muscle, with careful preservation of the facial nerve branches. A nerve stimulator may be employed intraoperatively to aid in the identification and protection of smaller, less visible branches; (**d**) Exposure of the subcondylar fracture under direct vision; (**e**) Open reduction and internal fixation using titanium miniplates and monocortical screws; (**f**) Skin closure; note the minimal scar and absence of swelling; (**g**) Preoperative 3D CT scan showing the displaced subcondylar fracture; (**h**) Postoperative 3D reconstruction demonstrating accurate reduction and stable fixation. This approach provides excellent surgical exposure with minimal risk of facial nerve damage, limited esthetic impact, and low complication rates.

The retromandibular approach provides reliable exposure and stable fixation for subcondylar mandibular fractures, with low overall morbidity. Comparing the anteroparotid and transparotid variants in a cohort of 80 consecutive patients, our findings add new evidence on how the choice of surgical corridor influences parotid-related complications while preserving favorable functional outcomes. Mandibular fractures remain among the most frequent traumatic injuries in the maxillofacial region, second only to orbital fractures in many trauma registries [1,9,10]. Their incidence is particularly high in young adult males and is often associated with interpersonal violence, road traffic accidents, and accidental falls [11]. Among these, subcondylar fractures represent a distinct subset due to their anatomical location, biomechanical implications, and functional consequences. The management of subcondylar fractures poses unique challenges, given the proximity to the temporomandibular joint (TMJ), the facial nerve, and the complexity of achieving stable occlusion and symmetric mandibular movement postoperatively. Accordingly, the management of subcondylar fractures is regarded as a fundamental surgical skill for oral and maxillofacial surgeons and has been identified as a quality marker of adequate specialty training within European educational standards [12]. Over the years, a wide variety of treatment protocols have been proposed, ranging from closed conservative techniques to open surgical approaches with internal fixation, each with specific indications, advantages, and drawbacks. The management of subcondylar mandibular fractures remains a subject of ongoing debate, particularly regarding the most appropriate surgical approach and fixation strategy. These fractures account for a substantial proportion of mandibular trauma, with estimates ranging from 25% to 35% of all mandibular fractures [6,13], and they carry both functional and aesthetic implications. While conservative management was historically preferred, recent evidence has increasingly supported open reduction and internal fixation (ORIF) in cases of displaced, bilateral, or unstable fractures due to superior outcomes in occlusal restoration, mandibular mobility, and prevention of long-term temporomandibular joint (TMJ) dysfunction [7,14]. Recent systematic reviews and biomechanical studies published between 2023 and 2025 further support the role of ORIF and retromandibular access in displaced subcondylar fractures [7,13]. In our study, all patients were treated via a retromandibular approach, using an anteroparotid or transparotid transmasseteric pathway (Figure 4). This access route provides direct visualization of the subcondylar region, allows for precise reduction, and enables stable fixation under direct vision. Compared with the transparotid route, the anteroparotid approach also minimizes the risk of minor complications such as sialocele formation, as the parotid gland is gently retracted without being traversed. Although 2 out of 26 patients who underwent a transparotid approach developed postoperative sialocele, no such cases occurred in the anteroparotid group. However, the difference was not statistically significant (*p* = 0.6510), likely due to the small sample size. Although both routes yielded comparable rates of facial nerve dysfunction and malocclusion, all cases of sialocele occurred after the transparotid approach, whereas no salivary complications were observed following the anteroparotid route. This supports the rationale for developing an anterior plane of dissection that preserves the integrity of the parotid parenchyma. These findings are consistent with the literature, where the retromandibular approach has been associated with favorable clinical outcomes, reduced operative time, and minimal scarring [7]. Koirala et al. [15], in a prospective clinical series, reported excellent fracture control and low complication rates using the transparotid variant, while other authors have highlighted the versatility of this approach even in comminuted or medially displaced fractures [8,16]. In our series, complication rates were low: transient facial nerve weakness occurred in 8% of patients, malocclusion in 6%, and visible scarring in 6%, with no cases of TMJ ankylosis or permanent facial nerve damage. Only 3% of patients still presented a visible scar at 6-month follow-up. These results compare favorably with previous reports, where transient nerve dysfunction is reported in 10–30% of cases depending on technique, dissection plane, and anatomical variability. Importantly, the risk of facial nerve injury is reduced when blunt dissection is performed in the correct avascular plane, as described in classic anatomical studies and recent refinements of the retromandibular approach [17,18]. The choice and configuration of fixation hardware also remains a topic of debate. While no universal standard exists, numerous studies have shown that double miniplate fixation offers greater biomechanical stability and fewer complications compared to single-plate constructs [19,20]. Our data support this observation: the use of two plates was associated with a significantly lower rate of postoperative complications (*p* < 0.05). Finite element analysis and cadaveric models confirm that dual-plate fixation, particularly in a triangular or trapezoidal configuration, minimizes rotational and torsional stress during early loading and mandibular function [8,21]. While three-dimensional plates and resorbable systems have shown promise in certain settings, titanium miniplates remain the most widely used solution due to their ease of application, cost-effectiveness, and predictability [19,22]. Another relevant finding from our analysis is the significant association between fracture complexity and operative time. Patients with bifocal or trifocal fractures required longer surgical procedures, which is consistent with prior studies emphasizing that increased fracture severity leads to more challenging reduction and longer intraoperative management. This increased complexity also potentially correlates with higher rates of malocclusion and esthetic issues, although our data did not reveal statistically significant associations in this regard. Alternative approaches such as the endoscopic-assisted intraoral technique have been explored, especially in light of their excellent esthetic outcomes and minimal risk to the facial nerve [23]. However, their wider adoption has been limited by technical difficulty, longer operative times, and limited access to the fracture site—particularly in cases involving displacement or comminution. In contrast, the retromandibular approach remains a reliable and reproducible option, particularly in centers with experience in facial trauma surgery. This study does present some limitations. First, its retrospective design introduces inherent bias, particularly in terms of selection and documentation. Second, the relatively small sample size and the absence of a control group treated through alternative surgical approaches limit the generalizability of our findings. Additionally, patient-reported outcome measures (PROMs), which could offer valuable insight into subjective esthetic and functional recovery, were not included. Finally, the lack of long-term follow-up beyond 3 months may underestimate late-onset complications such as TMJ stiffness or occlusal relapse. Despite these limitations, our findings support the use of the retromandibular approach as a safe and effective option for subcondylar fracture management. The low complication rate, short operative time, and reliable exposure of the fracture site suggest that this technique should be considered a standard option in cases requiring ORIF, particularly when the fracture pattern is complex or medially displaced. Future developments in the management of subcondylar mandibular fractures are likely to move toward increasingly personalized and technology-assisted surgical strategies. The growing availability of patient-specific implants (PSI), virtual surgical planning, and three-dimensional preoperative simulation may further improve reduction accuracy and fixation stability, particularly in complex or comminuted fracture patterns. In parallel, endoscopic-assisted techniques and image-guided navigation systems offer the potential to minimize surgical invasiveness while enhancing intraoperative visualization and precision. Emerging applications of artificial intelligence, including automated fracture classification and decision-support tools for approach and fixation selection, may further refine treatment planning. Together, these advances support a shift toward tailored, patient-specific management of subcondylar fractures, while reinforcing the need for surgical expertise and careful case selection.

## 5. Conclusions

The retromandibular approach represents a safe and effective option for the surgical management of subcondylar mandibular fractures. In our cohort, it provided excellent exposure of the fracture site, allowing for precise reduction and rigid fixation, with a low rate of complications and favorable functional outcomes. The use of two miniplates appeared to reduce the incidence of postoperative issues, supporting current evidence on the biomechanical advantages of dual-plate fixation. Moreover, fracture complexity was significantly associated with increased operative time, reinforcing the need for careful preoperative planning in multifragmented patterns. While these findings confirm the reliability of the retromandibular approach, future prospective studies with long-term follow-up and functional assessments are warranted to validate these results and further optimize treatment protocols.

## Figures and Tables

**Table 1:** Main demographic and etiopathological data.

Variable	Category	n (%)
**Sex**	Male	54 (67.5%)
	Female	26 (32.5%)
**Mean Age (years)**		41.9 (range: 18–72)
**Trauma Etiology**	Road traffic accident	30 (38%)
	Assault	30 (38%)
	Fall	20 (24%)
**Fracture Pattern**	Monofocal	25 (32%)
	Bifocal	35 (44%)
	Trifocal	13 (16%)
	Comminuted	7 (8%)
**Associated Fractures**	Subcondylar + symphysis	27 (34%)
	Isolated subcondylar	22 (28%)
	Subcondylar + parasymphysis	5 (6%)
	Subcondylar + symphysis + contralateral condyle	5 (6%)
	Subcondylar + contralateral condyle	6 (8%)
	Other combinations	15 (18%)

## Data Availability

The data supporting the findings of this study are available from the corresponding author upon reasonable request.
